# The Department of Modern Nursing

**Published:** 1914-07-11

**Authors:** 


### THE DEPARTMENT OF MODERN NURSING.*

### IV. West London Hospital.

The West London Hospital was founded in 1856, and has subsequently been several times enlarged in response to the demands of the dense population which has now gathered round it. The hospital has no medical school, but is a centre for post-graduate work. The opportunities which it offers for the training of nurses are undoubtedly enlarged by this circumstance. The lectures, classes, and observations continually taking place in the presence of the nurses in training are of a nature to interest them in the symptoms of the patients and impart incidentally a considerable amount of insight into the progress and treatment of various diseases. Intelligent nurses who have been well grounded in the elements of anatomy and physiology are likely to derive much benefit from assisting at the bedside during post-graduate instruction, and although much that passes is above the level of their knowledge the importance of many aspects of nursing is brought very forcibly under their notice through the additional attention bestowed on the cases. The hospital contains 160 beds. There are fifty nurses in training, ten sisters, night superintendent, matron, and assistant matron.

### The Nurses' Quarters.

A new Nurses' Home has long been imperatively called for, for the recent quarters of the staff, always inadequate from a modern standpoint, have long been outgrown. The whole amount required for the erection of the Nurses' Home, £12,000, and that for the purchase of a site has now been promised. Negotiations are in progress for the purchase of the latter, and plans will be prepared as soon as possible for the new buildings. Under the good management, which has obtained satisfactory results in the teeth of every obstacle, the training school will no doubt go forward and achieve real distinction when adequately housed.

### The Probationers' Course.

The age limit is twenty-two to thirty-two, and the term of engagement is for four years. The salaries are £6, £12, and £18; staff nurses, £24 to £30; sisters, £35 to £45.

After a trial month, which may be extended, the probationer is appointed on the staff, and during her first year attends courses of elementary anatomy and physiology given by the Assistant Matron. During the second and third years she attends lectures on Medical and Surgical Nursing once a week from members of the medical staff, who generously give their services for the nurses' benefit. Examinations are held at the conclusion of each course. From time to time special courses are arranged on gynæcology and other subjects for the more advanced nurses. During the fourth year lessons are given on sick-room cookery by an instructor from the Universal Cookery and Food Association. The ward work is learned under the sisters, and any gaps which may have occurred in their technical knowledge are noted and made good during the fourth year; the small size of the training school enables the Matron to keep a close eye on the opportunities enjoyed by each probationer, and in consultation with each individually she ascertains what is lacking in their full equipment of knowledge. The nurse is expected to co-operate with the Matron in getting a complete mastery over her work. There are three large wards containing twenty-six beds each, five containing twelve beds each, and one ward of twenty-one cots for children under seven years of age. In the large wards the full ward staff consists of the sister and staff nurse (fourth year), and three probationers in their first, second, and third years.

There is one theatre in which the Sister and two nurses are constantly employed, the latter remaining six months at a time. Very good opportunities are afforded also in the out-patient department for practice in theatre work and dressings. In this department a large number of minor operations take place daily, and about 100 dressings a day fall to the nurses. A sister and six nurses are continually employed here, and over 3,000 patients are seen weekly. All the nurses pass through this branch of training, although it is not always possible to secure each probationer six months' training in the general theatre.

### Holidays and Recreations.

The nurses are allowed two hours off duty every day, with two and a half on Sunday. Once during the week leave is allowed from 5.30 to 9.30, and one whole day is given once a month. They have three weeks' holiday in the year. All nurses who desire to attend the 6 A.M. celebration on Sunday are permitted to do so without asking leave, merely inscribing their name on the slate provided for that purpose. This is a very good arrangement. It cannot be said that much opportunity offers for general recreation indoors under existing circumstances. This will no doubt be rectified when the new Nurses' Home is in being.

Rigid economy is imposed by the exigencies of funds, but we understand that this is not allowed to stand in the way of providing liberally for the nurses so far as is possible. The commissariat is good and plentiful, but we regret that the cost of the nurses' board is not separated from that of the hospital. In the interests alike of economy and of the nurses' comfort the managers should know what is spent on housekeeping in the nurses' quarters. In constructing the new Nurses' Home it is highly important to arrange for a complete severance of accounts between this establishment and the hospital.

* Previous articles of this series appeared in The Hospital of May 16, May 30, and June 13.

